# Impact of mixed-height vegetation patches on energy loss in open-channel flow

**DOI:** 10.1038/s41598-025-94744-1

**Published:** 2025-04-01

**Authors:** Zixin Yang, Xiaonan Tang, Fengping Li, Jiahong Liu, Hao Wang, Jia Wang, Changhai Li, Baoshan Fan

**Affiliations:** 1 Key Laboratory of Groundwater Resources and Environment (Jilin University), Ministry of Education, Changchun, China; 2 Jilin Provincial Key Laboratory of Water Resources and Water Environment, Jilin University, Changchun, 130021 China; 3 College of New Energy and Environment, Jilin University, Changchun, 130021 China; 4 Department of Civil Engineering, Xi’an Jiaotong-Liverpool University, Suzhou, 215123 China; 5 State Key Laboratory of Simulation and Regulation of Water Cycle in River Basin, China Institute of Water Resources and Hydropower Research, Beijing, 100038 China; 6 Key Laboratory of River Basin Digital Twinning of Ministry of Water Resources, Beijing, 100038 China; 7 Engineering and Technology Research Center for Water Resources and Hydroecology of the Ministry of Water Resources, Beijing, 100038 China; 8 Research Center On Cold Region Engineering, Ministry of Water Resources, Changchun, 130061 China

**Keywords:** Hydrology, Hydrology

## Abstract

This study investigates the influence of riparian vegetation on energy losses in open-channel flow, focusing on channels partially covered by mixed-height vegetation patches, a common feature in natural rivers and canals. While previous research has primarily focused on flow resistance in fully vegetated channels, there has been limited attention to channels with unevenly distributed vegetation patches. To address this gap, we developed an innovative experimental approach to evaluate energy loss in channels with mixed-height vegetation patches under different submergence conditions. The experimental setup involved a channel partially covered with vegetation of varying heights, mimicking the natural, uneven distribution of vegetation patches. The results provided key insights into flow velocity distribution and turbulence intensity under these conditions. Furthermore, we introduced a standardized conceptualization method for the submergence ratio, specifically the concept of effective height (${h}_{e}$), to standardize the calculation methods for submerged and emergent vegetation.Using this parameter, we derived a theoretical formula for calculating energy loss caused by vegetation patches, which closely matched the experimental data. This method offers a reliable framework for calculating hydraulic resistance in channels with uneven vegetation distribution.

## Introduction

In recent years, climate change has increased the frequency of extreme rainfall events, thereby putting increasing pressure on urban river systems and coastal flood control infrastructure^1^. This has led to the study of energy losses associated with vegetation becoming a critical focus in hydrodynamic modelling and water resource management. Vegetation, as a key ecological component in hydrodynamic systems such as lakes, rivers, and tidal estuaries, not only influences the morphological evolution of these systems but also plays a crucial role in the interaction between flow dynamics and ecological processe^1,2^. In these environments, the flow exerts forces on vegetation, leading to branch deformation or root erosion, while the vegetation itself generates resistance to the flow^3,4^. This bidirectional interaction significantly affects water surface slopes, flow velocity distributions, and turbulence structures^5–7^. These hydrodynamic effects, in turn, influence vegetation community distribution and biological habitats^8,9^ Recent research further underscores the growing importance of vegetation in flood management and watershed ecological protection^10^.

Vegetation distribution is typically patch, with patches referring to spatially concentrated clusters of vegetation commonly found in natural river channels and other hydrodynamic systems^11^. These patches often exhibit irregularities in height and density, and their spatial heterogeneity exerts a complex influence on flow hydrodynamics, particularly regarding turbulence generation, energy dissipation, and flow resistance. This complexity complicates the accurate assessment of vegetation patch effects on flow resistance. In river environments, vegetation resistance plays a significant role in flood flow distribution, affecting floodwater levels and duration^12^. Understanding the interaction between vegetation and flow, especially the energy losses induced by vegetation, is essential for predicting flow resistance and the ecological trajectory of river and coastal vegetation systems. However, most existing studies assume uniform vegetation distribution and consistent vegetation height, disregarding the heterogeneity typically present in natural river systems. This assumption limits the understanding of vegetation resistance effects in real flow conditions.

A substantial body of research has been conducted to investigate the influence of vegetation on river hydraulics, utilizing a combination of laboratory measurements, field observations, and numerical simulations. In these investigations, vegetation is typically idealized as a rigid structure covering the bottom of the channel to simplify the analysis process^13^. Pasche and Rouvé^14^ first described the relationship between vegetation density and flow resistance, examining how vegetation influences open channel flow. Nepf^15^ started to study the dynamic mechanisms of flow resistance caused by vegetation. Baptist et al.^16^ formulated equations to quantify vegetation resistance, while Liu et al.^17^ studied regular combinations of vegetation with varying densities and heights. Antonino D’Ippolito et al.^18^ extended this work to investigate irregularly distributed vegetation with consistent heights. Although these studies have advanced the understanding of vegetation-water interactions, they usually assume that vegetation distribution and height are uniform and do not explicitly account for the energy losses caused by vegetation patches, which contrasts with the spatial heterogeneity observed in natural river systems.

To address these research gaps, this study introduces the concept of effective height (${h}_{e}$), a universal method for measuring vegetation height applicable to complex scenarios, derived by mathematical methods. In simple terms, it can standardize the submerged height of vegetation to determine the frontal area of vegetation patches. This refers to the area of vegetation projected onto a plane normal to the flow direction, which is a key parameter for quantitatively evaluating the morphology of vegetation patches. Furthermore, this parameter is also applicable to flexible vegetation. This is because, regardless of how flexible vegetation tilts under the influence of flow, as long as the frontal area of the flexible vegetation is determined, resistance can be calculated similarly to a rigid cylindrical structure. To investigate this, a series of controlled experiments were conducted in an inclined flume to simulate various conditions for vegetation coverage with different height combinations and distributions.

In the experiments, the water flume is partially covered with vegetation of varying heights, while the rest is bare. To simulate the natural morphology of vegetation patches, short vegetation was placed near the center of the flume while the tall vegetation was near the wall. By adjusting the flow rate and water depth, velocity profiles, turbulence intensity, and energy gradients were measured under fully submerged, partially submerged, and outflow conditions. The experimental results demonstrated the complex effects of heterogeneous vegetation patch distribution on flow field characteristics. Based on these experimental data and literature reviews, this study derived and validated a theoretical formula for flow energy dissipation caused by vegetation patches.

The method for calculating the resistance of vegetation patches proposed in this study can predict the impact of uneven vegetation patch distribution on flow resistance under close-to-natural conditions, offering greater usability and reliability. This research not only deepens the understanding of vegetation patch-flow interactions but also provides practical tools for managing natural rivers and designing man-made channels. The parameters and calculation methods presented may be incorporated into hydrological models to enhance the accuracy of hydrodynamic simulations. This integration will provide valuable insights for policy-making in key areas, including water resource management, sediment control, and the preservation of healthy aquatic ecosystems. Additionally, this approach can be used to assess the effects of varying vegetation configurations on flow dynamics and sediment transport, thereby aiding decision-making in river restoration and floodplain management. It may also be applied in the design of flood protection strategies, where it plays a pivotal role in modulating flow velocity and turbulence, thus influencing flood propagation. Finally, by incorporating the concepts of energy loss and flow resistance into ecological models, the findings of this study can inform efforts for riparian zone restoration, enhancing biodiversity, improving habitat quality, and contributing to the sustainable development of aquatic ecosystems.

## Materials and methods

### Experimental apparatus

**Fig. 1:**
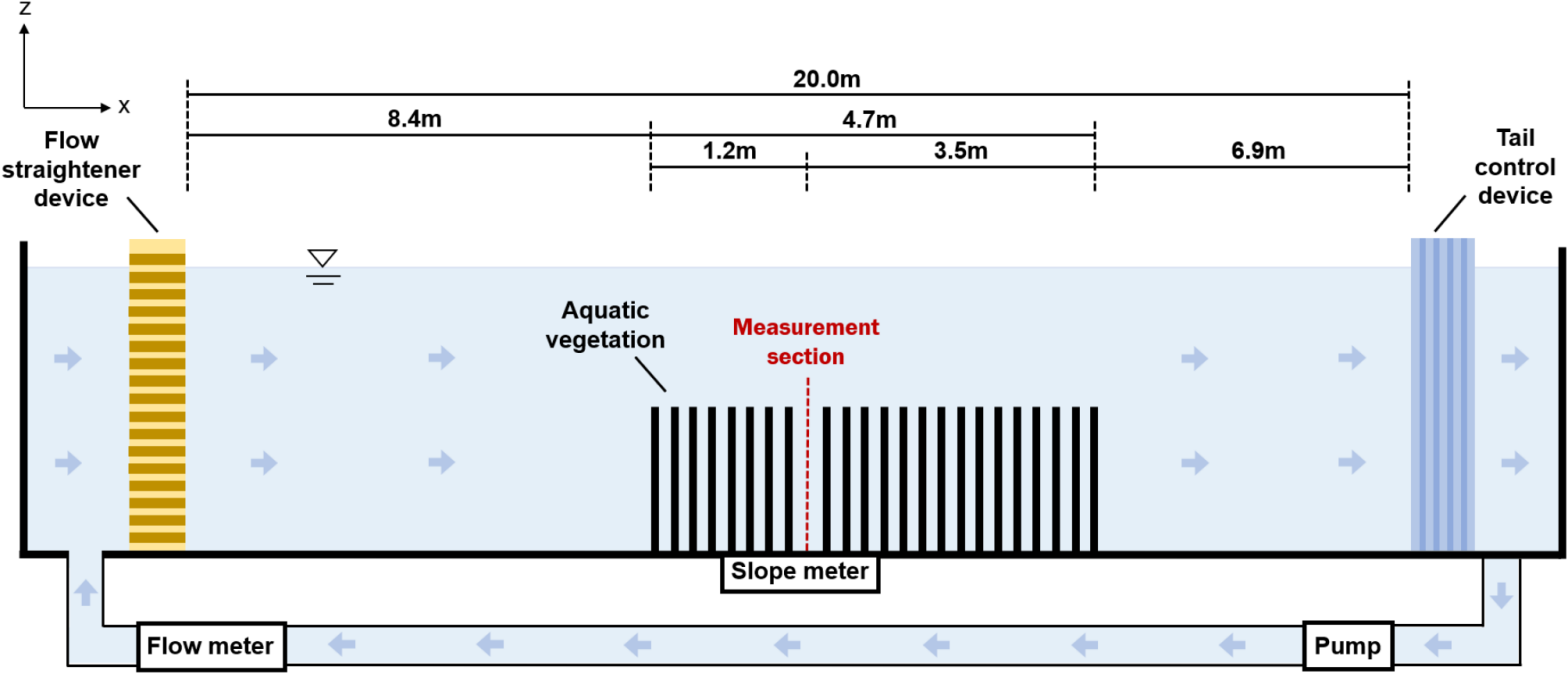
Sketch of the experimental flume.

The experiment was conducted in a water-recirculating flume with a rectangular cross-section, located in the Fluid Mechanics Laboratory at Xi’an Jiaotong-Liverpool University (XJTLU) in Suzhou, China. The experimental setup consisted of a rectangular water tank at the inlet, an outlet flow control tailgate, flow straightening devices, a pump, a slope meter, and an electromagnetic flow meter, as shown in Fig. 1. The flume is 20 m in length and 0.4 m in width, with reinforced glass used for both the bottom and walls. The slope of the flume is adjustable via an electric motor, with the tilting angle displayed on the slope meter. This study fixed the bed slope ($i$) at 0.003. The water depth is controlled by the inlet pump and tailgate, while the flow rate is measured using the electromagnetic flow meter.

This experiment utilized two flow velocity measurement devices: a micro 3D Nortek Acoustic Doppler Velocimeter (ADV) and a mini propeller velocimeter. The ADV is frequently employed in laboratory environments to measure three velocity components, thereby enabling the analysis of turbulence characteristics. It is especially effective in high-shear conditions, near boundaries, under breaking waves, and in various other applications^19^. Propeller velocimetry is commonly employed to measure the streamwise mean velocity in both laboratory and field applications of open channel flow. This method operates on the principle that the flow of water drives the rotation of the meter’s blade. As the flow rate increases, the rotational speed of the blade also increases, with the number of rotations recorded over a defined time interval. Equation (1) demonstrates the relationship between the average streamwise velocity and the rotational count.

1 $$\begin{array}{c}V=K\frac{n}{t}+C\end{array}$$

where $V$ is the velocity at the measurement point, $C$ is the intercept of the calibration coefficient, $K$ is the slope of the calibration coefficient, $t$ is measurement duration, and $n$ is count number. $K\hspace{0.17em}$= 1.78 and $C\hspace{0.17em}$= 0.88 for this measurement.

### Vegetation arrangement

**Fig. 2:**
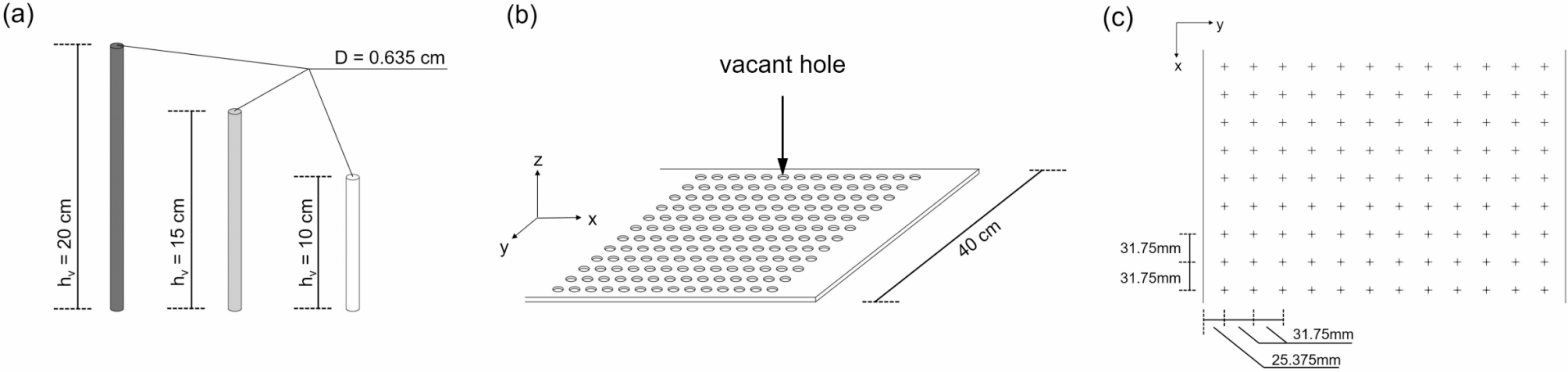
Configuration of vegetation: (**a**) Rod size, (**b**) Bottom plate, (**c**) Bottom plate layout.

In this experiment, vegetation was simulated using rigid rods representing three distinct heights: 10 cm, 15 cm, and 20 cm. All rods had a consistent diameter of 0.635 cm ($D$), as shown in Fig. 2a. A 40 cm wide, pre-perforated plastic plate was placed at the bottom of the flume to secure the rods, as illustrated in Fig. 2b,c. This arrangement facilitated the creation of various vegetation patterns, as required.

### Experiment conditions

**Fig. 3:**
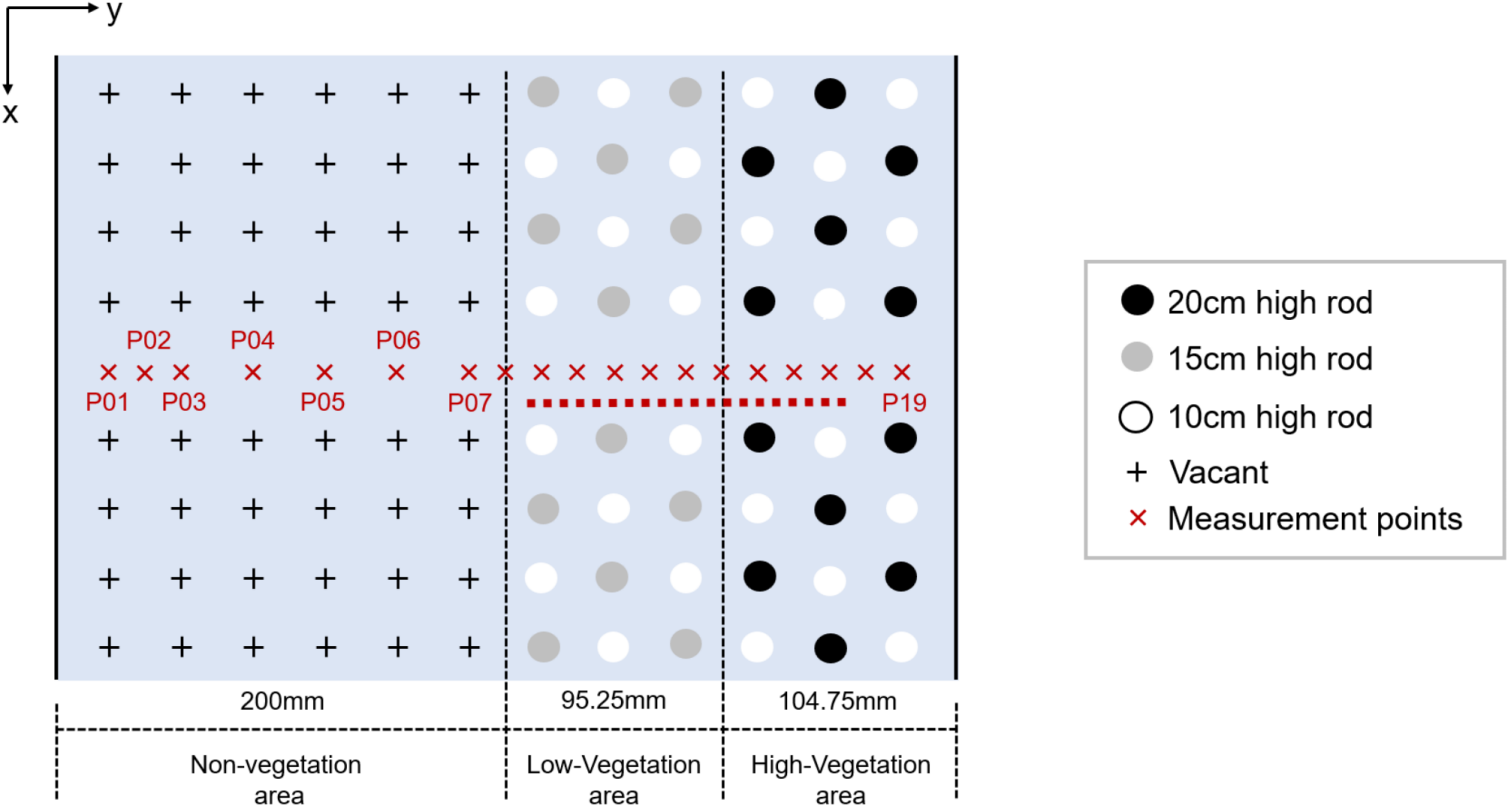
Experimental setup diagram with the cross-sectional division for vegetation patches.

The aquatic vegetation patches, simulated using rigid rods, span a 4.7 m section of the flume, starting 8.4 m from the entrance. The measurement section is located 1.2 m downstream from the start of the vegetation-covered area (Fig. 1). The bed of the flume is partially vegetated, with mixed vegetation covering half of its width, as shown in Fig. 3. This setup creates a partially vegetated channel: one half is devoid of vegetation, while the other half is covered by mixed vegetation. The vegetation coverage has a width (${B}_{v}$) of 20 cm and a length (${L}_{v}$) of 470 cm.

To examine the effect of vegetation on flow under varying flow depths, three experimental conditions were established: non-submerged, partly-submerged, and fully-submerged, designated as Test 1, Test 2, and Test 3, respectively. These conditions were achieved by setting flow depths of 10–15 cm, 15–20 cm, and greater than 20 cm. For analytical purposes, the experimental area was divided into three sections: Non-vegetation, Low-vegetation, and High-vegetation. The flow conditions for each test are summarized in Table 1.

**Table 1:** A summary of experiment flow conditions.

Parameters	Test 1	Test 2	Test 3
Water depth $H$ (cm)	11.59	17.23	24.49
Discharge $Q$ (L/s)	8.98	12.46	17.85
Reynolds number $Re$	18,509	21,792	26,124
Froude number $Fr$	0.182	0.139	0.118
Submerged condition	Emergent	Part-submerged	Full-submerged

The Reynolds number ($Re$) and Froude number ($Fr$) for each test, as presented in Table 1, were calculated using Eqs. (2) and (3), respectively. All experiments were conducted under turbulent and subcritical flow conditions, as shown in the table.

2 $$Re=\frac{\rho vR}{\mu }$$

where $\rho$ is the fluid density; $v$ is the cross-sectional velocity; $R$ is the hydraulic radius; $\mu$ is the dynamic viscosity;

3 $$Fr=\sqrt{\frac{{v}^{2}}{gH}}$$

where $g$ is the gravitational acceleration; $H$ is the water depth.

### Measurement section

The measurement location was situated 1.2 m downstream from the beginning of the vegetation section, as illustrated in Fig. 1. In the lateral direction, velocity measurements were taken at multiple points, with a higher density of measurements in the vegetated zone compared to the non-vegetated zone. A total of 19 measurement positions, labeled P01 to P19, were distributed across the section, as shown in Fig. 3. At each position, velocities were recorded at various heights to construct a vertical velocity profile. The number of vertical measurement points varied with the flow depth, with more points typically measured near the bed and at the top of the vegetation to capture potential large velocity gradients.

## Analytical method

The study utilized a range of parameters to conduct a thorough analysis, with each parameter chosen based on its relevance to the research objectives. These parameters are detailed in the subsequent sections, along with concise descriptions for clarity and context.

### Turbulence intensity

Turbulence intensity is a crucial parameter that quantifies the degree of disturbance in the outer flow^20^. The turbulence characteristics at the observation point are statistically represented by the instantaneous velocity *u*, as defined in Eq. (4).

4 $$u=\overline{u }+{u}{\prime}$$

where $u^{\prime }$ is the fluctuating velocity, $\overline{u }$ is the time-averaged velocity. Statistically, the mean of the fluctuating velocity $\overline{{u^{\prime } }}$ is zero. The turbulence strength is quantified using the root mean square of the velocity fluctuations, known as the fluctuating velocity intensity, as defined in Eq. (5)^21^.

5 $$\sqrt {\overline{{u^{\prime 2} }} } = \sqrt {1/3\left( {\overline{{u_{x}^{\prime 2} }} + \overline{{u_{y}^{\prime 2} }} + \overline{{u_{z}^{\prime 2} }} } \right)}$$

where $u_{x}^{\prime } \;u_{y}^{\prime } \;u_{z}^{\prime }$ are the fluctuating velocity components in the x, y, z directions, respectively.

In practical applications, the dimensionless root mean square velocity, denoted as $\frac{{\sqrt {\overline{{u^{\prime 2} }} } }}{{\overline{U}}}$, is commonly referred to as turbulence intensity or relative fluctuating velocity intensity. This parameter is widely used to quantify the turbulence of a flow. The corresponding parameter $\overline{U }$ is mathematically defined in Eq. (6).

6 $$\overline{U }=\sqrt{{\overline{{u }_{x}}}^{2}+{\overline{{u }_{y}}}^{2}+{\overline{{u }_{z}}}^{2}}$$

where $\overline{{u }_{x}}$, $\overline{{u }_{y}}$, $\overline{{u }_{z}}$ are the time-averaged velocity in the x, y, z directions, respectively, and $\overline{U }$ is their combined velocity in three directions.

### Velocity gradient

To gain a deeper understanding of the effect of vegetation on turbulent flow intensity and investigate the relationship between turbulent intensity and velocity, the turbulent intensity at each measurement point is compared with the velocity gradient between adjacent points. The velocity gradient is calculated using Eq. (7).

7 $${g}_{u}=\frac{\overline{{u }_{1}}-\overline{{u }_{2}}}{\Delta d}$$

where ${g}_{u}$ is the velocity gradient, $\overline{{u }_{1}}$ is the time-averaged velocity at the measurement point, $\overline{{u }_{2}}$ is the time-averaged velocity at the adjacent measurement point, and $\Delta d$ is the distance between two measurement points.

### New theoretical method for calculating the energy loss of vegetation patches

**Fig. 4:**
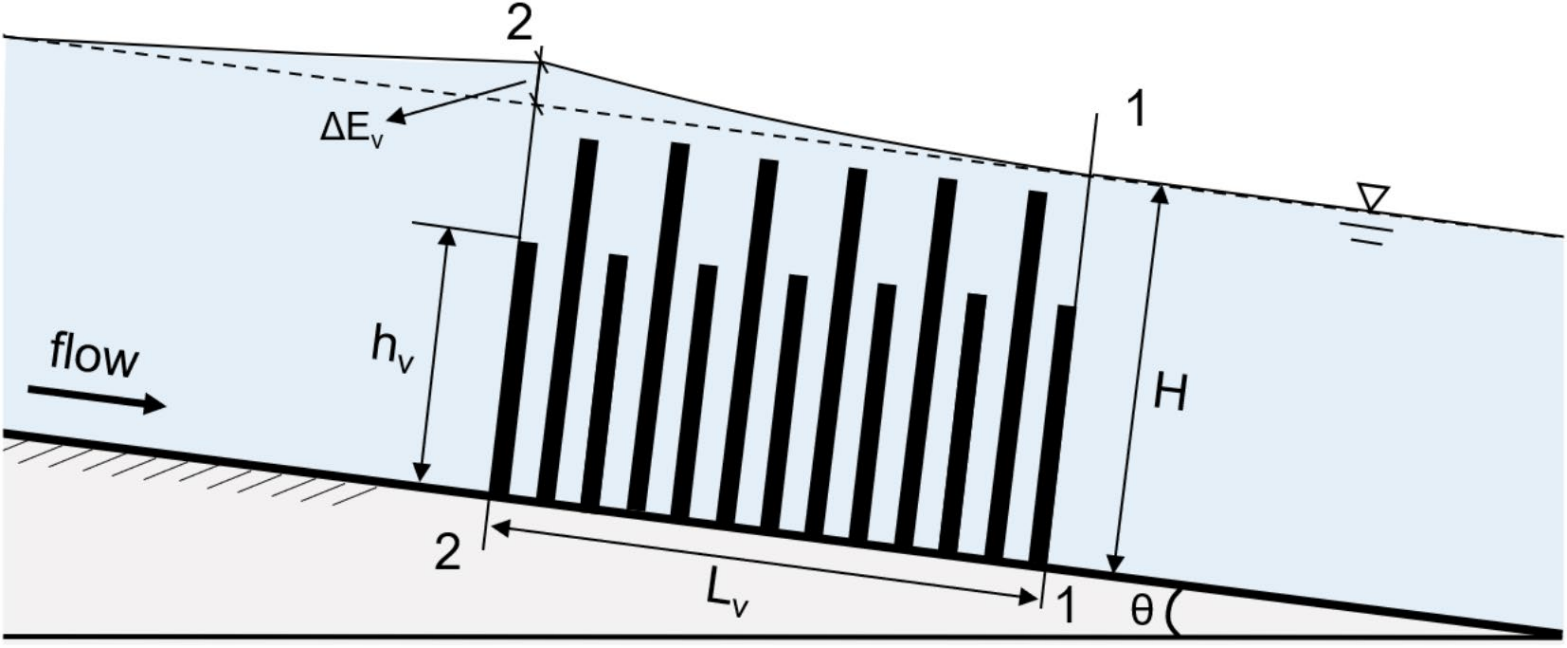
A sketch of open-channel flow with mixed-height rigid vegetation.

The presence of a vegetation patch in open channels introduces complexity in calculating energy loss, especially when the vegetation is submerged or non-submerged. In these channels, the upper and lower limits of the vegetation section are designated as the 1–1 and 2–2 sections, respectively (Fig. 4). When the head loss ($\Delta {E}_{v}$) induced by vegetation is known, the hydraulic gradient can be determined using the energy equation, as follows:

8 $${z}_{1}+{h}_{1}+{\alpha }_{1}\frac{{V}_{1}^{2}}{2g}={z}_{2}+{h}_{2}+{\alpha }_{2}\frac{{V}_{2}^{2}}{2g}+{h}_{f}+{h}_{j}+\Delta {E}_{v}$$

where, ${z}_{1}$, ${h}_{1}$, ${V}_{1}$ and ${z}_{2}$, ${h}_{2}$, ${V}_{2}$ represent the water head, depth, and velocity at the upstream and downstream sections, respectively. ${\alpha }_{1}$ and ${\alpha }_{2}$ are the velocity coefficients of cross-section. In this study, a constant value of 1.05 is used for the calculation^22^. ${h}_{f}$ and ${h}_{j}$ denote the frictional and local head losses. In this experiment, under quasi-uniform conditions, the water surface in the non-vegetated flow section is parallel to the bed slope. As a result, the effects of ${h}_{f}$ and ${h}_{j}$ are neglected.

The distance ${L}_{v}$ between cross-sections 1-1 and 2-2 represents the length of the vegetation channel. The relationship between the vegetation energy gradient ${J}_{a}$ and head loss $\Delta {E}_{v}$ can be expressed as:

9 $$\Delta {E}_{v}={J}_{a}{L}_{v}$$

The key to calculating the energy loss in vegetated channels, as shown in Fig. 4, is to determine the vegetation energy gradient (${J}_{a}$). Cheng^23^ introduced the concept of the submerged vegetation-obstructed hydraulic radius and developed a single-layer model to estimate ${J}_{a}$, applicable to both flexible and rigid vegetation. For emergent vegetated flows, D'Ippolito et al.^18^ conducted an extensive review of drag coefficient formulas in open channels. Their analysis, based on 29 subcritical flow profiles from flume experiments with emergent vegetation, revealed that certain formulas significantly underestimated flow depths.

To standardize the calculation of vegetation under different submerged conditions, we defined ${h}_{e}$ as the effective height. This parameter provides a consistent measure of vegetation height across different submergence levels within a single experiment. Specifically, ${h}_{v}$ represents the height of vegetation, as shown in Fig. 2a.

10 $${h}_{e}=\left\{\begin{array}{c}{h}_{v} ; {h}_{v}<H\\ H ; {h}_{v}>H\end{array}\right.$$

The flow resistance of individual vegetation is given by:

11 $${F}_{D}=\frac{{C}_{D}}{2}{Dh}_{e}\rho {v}_{v}^{2}$$

In this context, ${F}_{D}$ represents the drag force exerted on vegetation per unit area. The resistance coefficient ${C}_{D}$ is greater than 0.5^24^. For Reynolds numbers within the range 150 ≤ Re ≤ 10^5^, ${C}_{D}$ is approximately 1.2. For 10^5^ ≤ Re ≤ 3.5 × 10^6^, ${C}_{D}$ ranges from 0.2 to 1.2^25^.

12 $${F}_{Dm}=\sum_{i=1}^{m}\frac{{C}_{D}}{2}{{D}_{i}h}_{ei}\rho {v}_{vi}^{2}/({B}_{v}{L}_{v})$$

where ${B}_{v}$ represents the vegetation coverage width, ${L}_{v}$ is the vegetation coverage length, and ${v}_{v}$ is the flow velocity through the vegetation. The additional flow resistance due to the vegetation in the channel is calculated per unit bed area, while ignoring resistantce in non-vegetated areas.

To simplify Eq. (12), we approximate the vegetation patch of different heights as a patch of same height by using the average effective height, ${h}_{em}$, as the height of the patch:

13 $${F}_{Dm}=\frac{{C}_{D}}{2}\frac{{Dh}_{em}}{{B}_{v}{L}_{v}}{\rho U}_{v}^{2}$$

where ${h}_{em}$, the average effective height of the patch, is defined as Eq. (14) ($m$ represents the number of vegetation), and ${U}_{v}$ is the velocity through the vegetation (bulk velocity), defined as (15)

14 $${{h}_{em}=\frac{1}{m}\sum_{i=1}^{m}{h}_{ei}}$$

15 $${U}_{v}\approx \frac{1}{m}\sum_{i=1}^{m}{v}_{vi}$$

${\phi }_{v}$ is the volume concentration of vegetation, defined as the percentage of the vegetation volume per unit volume of water flow. With the effective height ${h}_{e}$ defined in the previous section, ${\phi }_{v}$ can be expressed as:

16 $${\phi }_{v}=\sum_{i=1}^{m}\frac{\pi {D}_{i}^{2}}{4}\frac{{h}_{ei}}{H}/({B}_{v}{L}_{v})$$

Consider a rectangular section where steady and locally uniform flow conditions require a local force balance between the flow’s body force and the drag term. Wang et al.^26^ derived the relationship between flow resistance and the vegetation energy gradient ${J}_{a}$:

17 $$\gamma H\left(1-{\phi }_{v}\right){J}_{a}={F}_{Dm}$$

where $\gamma$ represents the specific weight of the fluid.

**Fig. 5:**
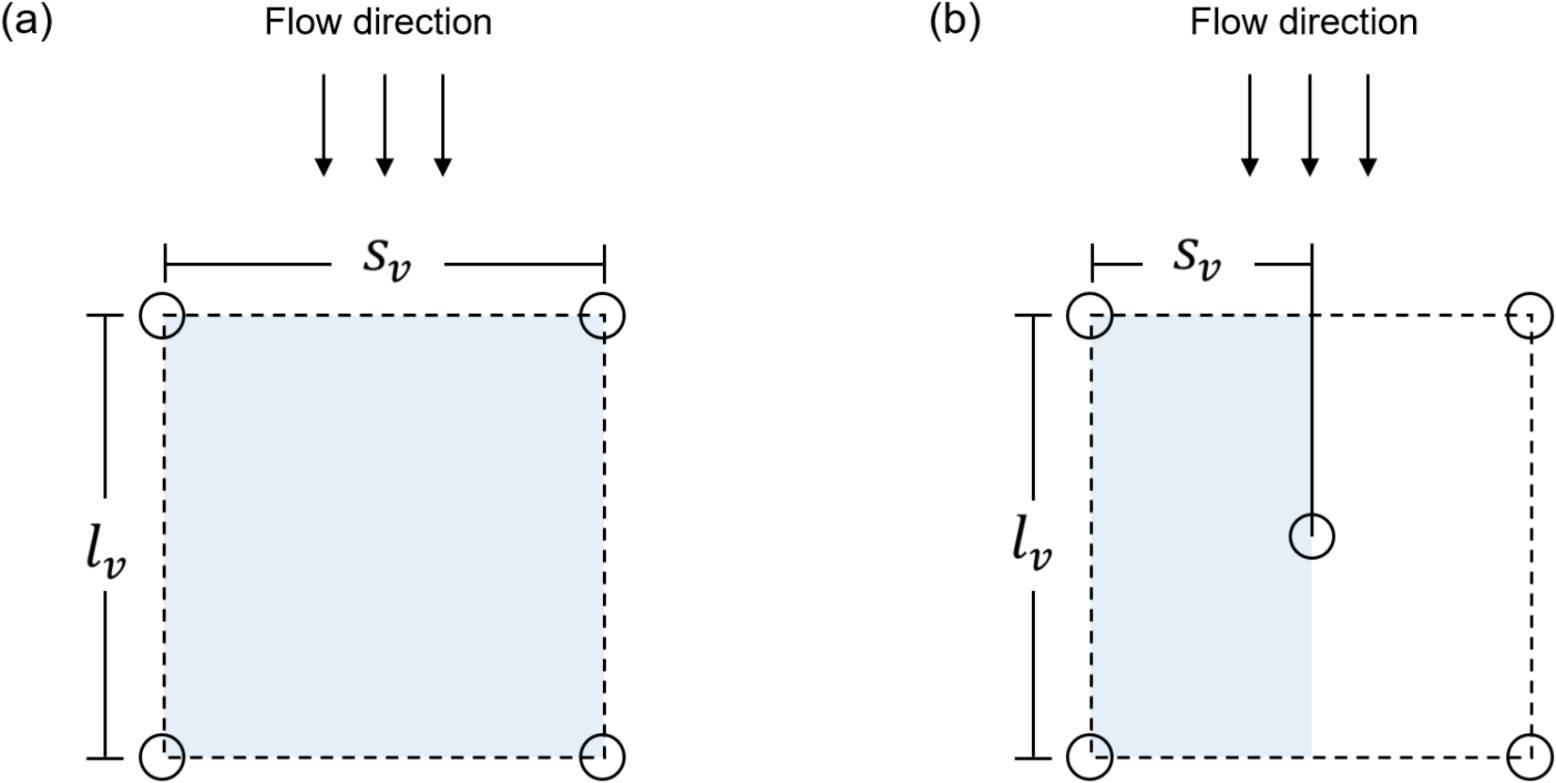
Calculation method schematic: (**a**) 4-point (**b**) 5-point.

Most research on vegetation resistance has concentrated on vegetation with uniform height and distribution. In contrast, riverbank vegetation in natural rivers typically forms canopies, where the height of the vegetation varies within each patch. To theoretically evaluate the resistance and energy loss associated with a vegetation patch, we propose novel methods for calculating ${J}_{a}$. We represent the vegetation distribution within a patch using two configurations: 4-point and 5-point, as shown in Fig. 5a,b, respectively. In these configurations, the gap between dowels in the flow direction is denoted as ${l}_{v}$, the gap perpendicular to the flow direction as ${s}_{v}$, and each dowel occupies an area of ${l}_{v}\times {s}_{v}$.

18 $${F}_{Dm}=\frac{{C}_{D}}{2}D{h}_{em}\rho {U}_{v}^{2}/({l}_{v}{s}_{v})$$

By substituting Eq. (18) into Eq. (17), the vegetation energy gradient ${J}_{a}$ is obtained as Eq. (19), where $\frac{{h}_{em}D}{{l}_{v}{s}_{v}}$ represents the superficial area of the vegetation stems per unit bed area of the vegetation patch, which can be interpreted differently depending on the vegetation distribution type.

19 $${J}_{a}=\frac{{C}_{D}}{2}\frac{1}{1-{\phi }_{v}}\frac{{h}_{em}D}{{l}_{v}{s}_{v}}\frac{{U}_{v}^{2}}{gH}$$

Considering the absence of significant lateral motion in one-dimensional flow, i.e., no lateral surface slope within a cross-section, which is established at the end of the patch, the energy consumption (or power) per unit flow remains the same^27^. This can be expressed as: $\frac{{U}_{v}^{2}}{2g}\cdot \frac{1}{{B}_{v}{U}_{v}}=\frac{{U}^{2}}{2g}\cdot \frac{1}{BU}$. Therefore, the average flow velocity over the vegetation patch is:

20 $${U}_{v}=\frac{{B}_{v}}{B}U$$

where $U$ is the cross-sectional average velocity, ${B}_{v}$ is the vegetation cross-sectional width (as shown in Fig. 3, and $B$ is the total width of the water flume.

Since $\frac{1}{1-{\phi }_{v}}\approx 1$, Eq. (19) simplifies to:

21 $${J}_{a}=\frac{{C}_{D}}{2}\frac{{h}_{em}D}{{l}_{v}{s}_{v}}{\left(\frac{{B}_{v}}{B}\right)}^{2}\frac{{U}^{2}}{gH}$$

The right-hand side of Eq. (21) consists of four terms: $\frac{{C}_{D}}{2}$, $\frac{{h}_{em}D}{{l}_{v}{s}_{v}}$, ${\left(\frac{{B}_{v}}{B}\right)}^{2}$, and $\frac{{U}^{2}}{gH}$, each representing a different factor affecting the energy gradient ${J}_{a}$. The term $\frac{{C}_{D}}{2}$ is one-half of the drag coefficient, $\frac{{h}_{em}D}{{l}_{v}{s}_{v}}$ was discussed above, ${\left(\frac{{B}_{v}}{B}\right)}^{2}$ represents the square of the ratio of the vegetation patch width to the total width of the river reach, and $\frac{{U}^{2}}{gH}$ is the Froude number ($Fr$). By calculating ${J}_{a}$ using Eq. (21), the energy loss in Eq. (9) can be determined, thus solving the energy loss caused by the vegetation patches.

## Results and discussion

**Fig. 6:**
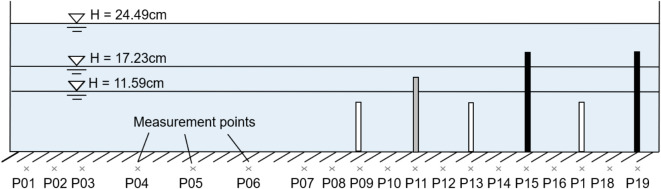
Overall description of experiments: Relationship between three water depths and vegetation height.

This section investigates the impact of vegetation on the hydrodynamic characteristics of the river, with a focus on flow velocity, turbulence intensity, and flow resistance at multiple measurement locations. Experimental data from three distinct water depths—labeled as Test 1, Test 2, and Test 3—were analyzed. The overall flow parameters are summarized in Table 1, and Fig. 6 illustrates the specific measurement locations within the vegetated area for all 3 tests.

### Velocity distribution

#### Vertical velocity

**Fig. 7:**
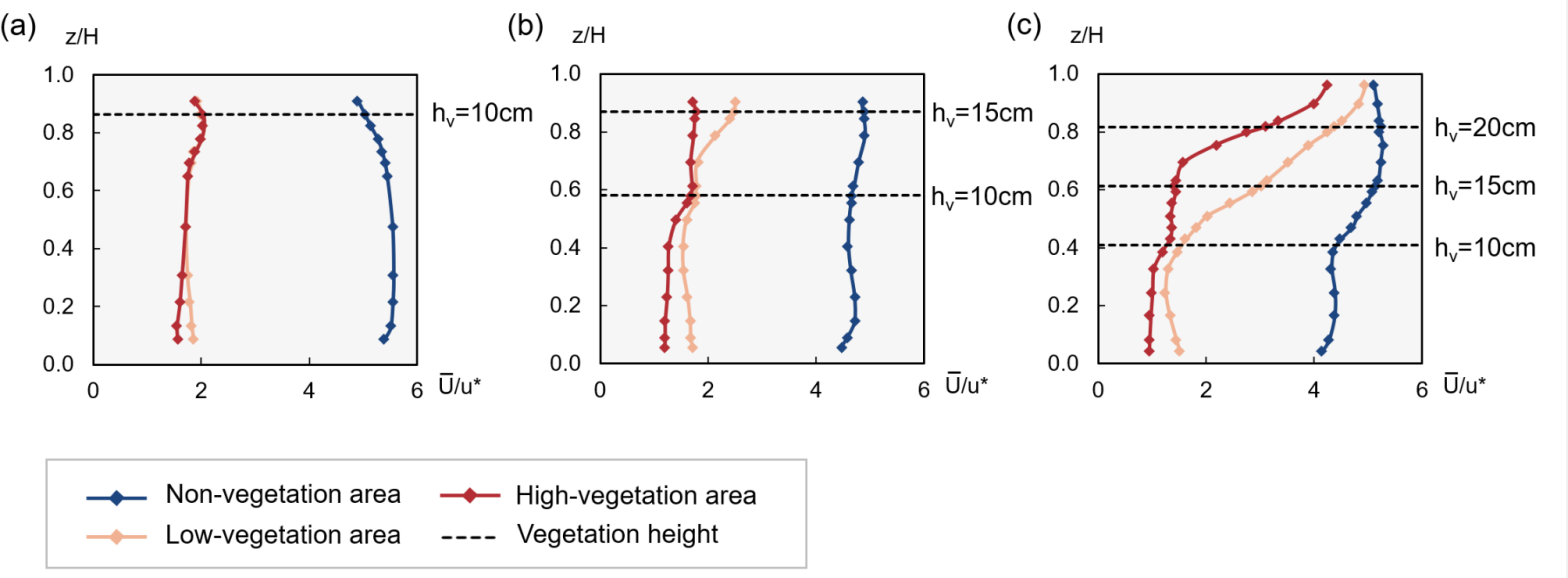
Mean velocity profiles of each region for (**a**) Test 1 (11.59 cm), (**b**) Test 2 (17.23 cm), and (**c**) Test 3 (24.49 cm).

Figure 7 shows the mean velocity profiles for the sub-sections of non-vegetation, low-vegetation, and high-vegetation areas for the three experimental scenarios. In the figure, $z$ is the vertical distance from the bed, $H$ is the water depth, and ${u}^{*}$ is the shear velocity, calculated as $\sqrt{gHJ}$, where $J$ is the surface slope. The dashed line in the figure indicates the vegetation height.

In general, the velocity in the non-vegetation area is significantly higher than in the vegetated areas (Fig. 7), with the velocity difference decreasing as water depth increases. Specifically, in the fully-submerged case (Test 3), the velocity difference among the vegetation areas was greatly reduced near the water surface (Fig. 7c).

For the vertical velocity profiles in Tests 1 and 2, a notable increase in velocity was observed at the top of the vegetation in the vegetated areas. Notably, the velocity increase at the top of the 10 cm-high vegetation was considerably greater than that at the top of the 15 cm-high vegetation (Fig. 7a,b). This effect is likely due to vegetation density: the 10 cm-high vegetation, with the highest density, generates the greatest resistance to flow, leading to the most significant velocity change at its top. This phenomenon is most pronounced in the high-vegetation area.

In the non-vegetated area, a comparison of the results from the three experimental scenarios shows that the vertical velocity change is relatively small. However, this change does not follow the logarithmic or power-law behavior commonly observed in open channel flow without vegetation, particularly in the region below the top of the 10 cm simulated vegetation, where the velocity remains almost constant. This observation is consistent with the findings of Rahimi et al.^28^. The phenomenon is most pronounced under emergent flow conditions (Test 1: Fig. 7a). As flow depth increases, as shown in Tests 2 and 3 (Fig. 7b,c), the velocity in the non-vegetated zone is increasingly influenced by the adjacent low-vegetation zone. This interaction leads to a slight increase in velocity, especially in areas with lower vertical vegetation density. These results align with the experimental findings of Changjun et al.^29^ At lower water depths, in Test 1 (Fig. 7a), the velocity profiles for the high-vegetation and low-vegetation areas almost overlap. As the water depth increases to 17.23 cm (Test 2) and even 24.49 cm (Test 3), although the difference in velocity increases between the high-vegetation and low-vegetation areas, the shapes of the two velocity profiles remain similar. This indicates that as long as the Reynolds number of the channel is greater than 500, the effect of vegetation on the flow properties is consistent^22^.

#### Lateral variation of layered-average velocity

**Fig. 8:**
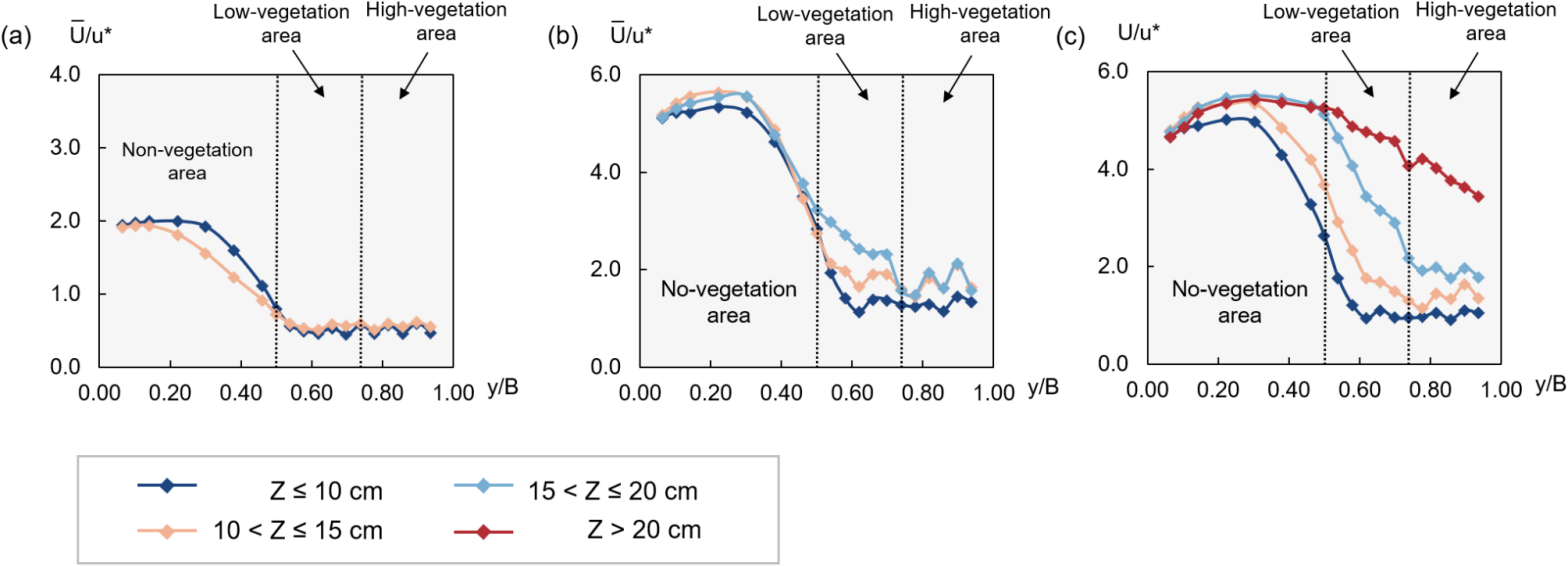
Lateral velocity distribution for (**a**) Test 1, (**b**) Test 2, and (**c**) Test 3.

To examine lateral velocity variation at different heights, the velocity data are divided into four layers: z ≤ 10 cm, 10 < z ≤ 15 cm, 15 < z ≤ 20 cm, and z > 20 cm. The average velocity for each layer in Tests 1-3 is shown in Fig. 8a–c, where y is the lateral position, $B$ is the total flume width, and ${u}^{*}$ denotes the shear velocity.

With a notable reduction in velocity in the vegetated areas compared to the non-vegetation area, the retarding effect of vegetation on velocity is more pronounced in the lower layer (z ≤ 15 cm) and decreases as the flow height increases. This is attributed to the higher resistance generated by the dense vegetation in the lower layer, which results in a lower velocity. Furthermore, within the vegetated areas, the velocity directly behind the vegetation is lower than the velocity observed behind the gaps between vegetation. This observation is consistent with the findings of Changjun et al.^29^ and Kumar et al.^30^.

This result shows the blocking and shedding effects of vegetation on flow. The velocity difference diminishes as the flow depth increases or progresses into higher layers.

### Turbulence intensity

**Fig. 9:**
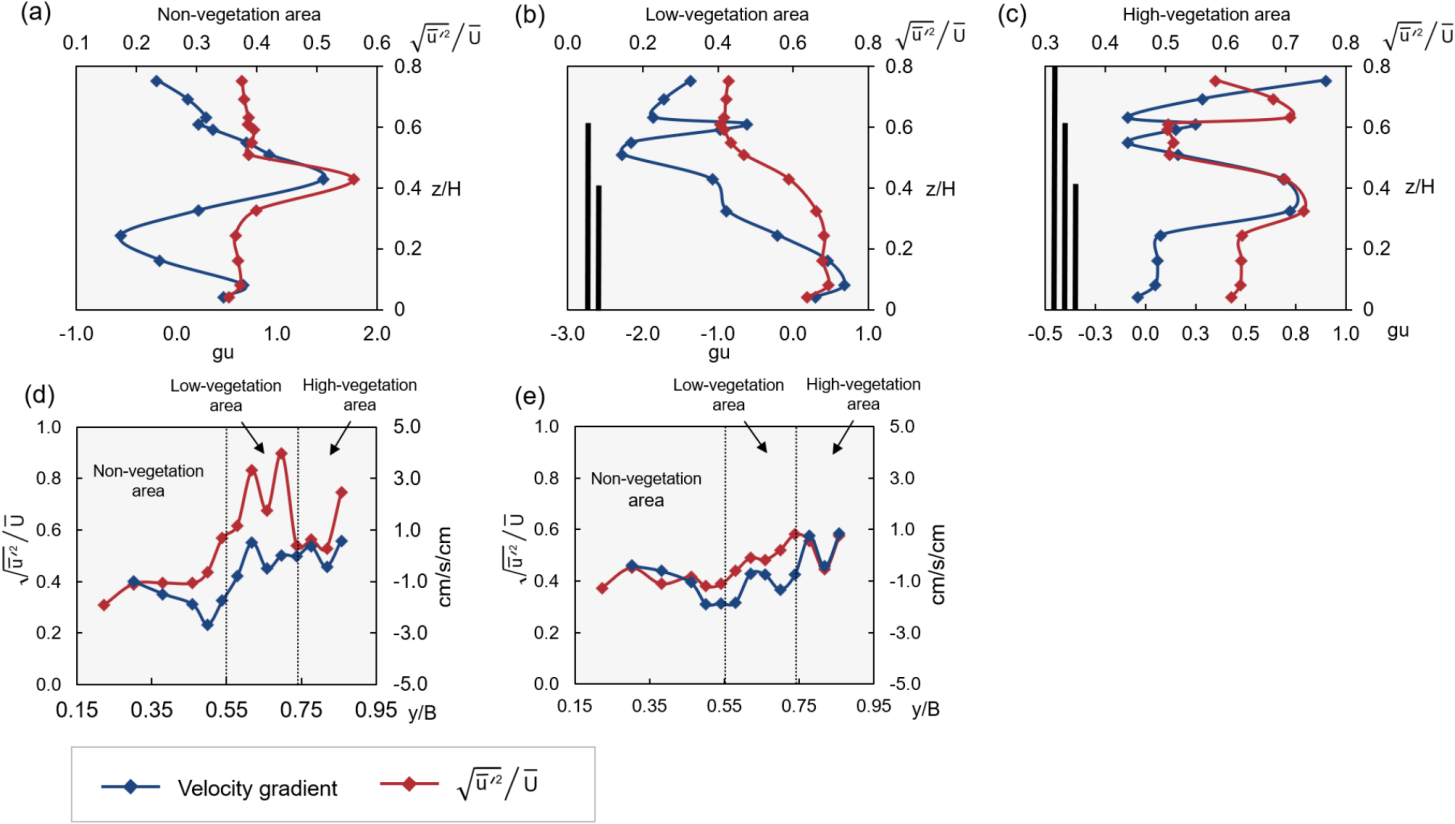
Turbulence intensity and velocity gradient distribution: (**a**–**c**) Vertical, (**d**, **e**) Lateral.

To assess the effect of vegetation on flow turbulence intensity, the relationship between turbulence intensity and velocity gradient is explored using Eq. (7). Turbulence intensity at each point is compared with the velocity gradient between neighboring points. Data from Test 3 ($H\hspace{0.17em}$= 24.49 cm) are shown in Fig. 9. The measurement section is divided into two areas (z < 10 cm and z > 10 cm), with weighted mean values of $\frac{{\sqrt {\overline{{u^{\prime 2} }} } }}{{\overline{U}}}$ and velocity gradient in each area were calculated separately. Results are shown in Fig. 9a–e, respectively.

In the vertical variation, as shown in Fig. 9b,c, in the area with vegetation, both velocity gradient and $\frac{{\sqrt {\overline{{u^{\prime 2} }} } }}{{\overline{U}}}$ appear to increase significantly near the top of the vegetation. Moreover, in the non-vegetation area, as shown in Fig. 9a, $\frac{{\sqrt {\overline{{u^{\prime 2} }} } }}{{\overline{U}}}$ and the velocity gradient appear to increase to some extent at the top of the short vegetation height, influenced by the Low-vegetation area. The velocity gradient has almost the same trend compared to $\frac{{\sqrt {\overline{{u^{\prime 2} }} } }}{{\overline{U}}}$, but the magnitude of the change is slightly different.

In the non-vegetation area, both $\frac{{\sqrt {\overline{{u^{\prime 2} }} } }}{{\overline{U}}}$ and the velocity gradient remain relatively low. In contrast, within the vegetated areas, both metrics show an increasing trend, which subsequently decreases with increasing measurement height, as illustrated in Fig. 9. Near the flume bed, at measurement positions where z < 10 cm, the values of $\frac{{\sqrt {\overline{{u^{\prime 2} }} } }}{{\overline{U}}}$ and the velocity gradient in the low-vegetation area significantly increase compared to the non-vegetation area, reaching their maximum values. However, these values decrease in the high-vegetation area, as depicted in Fig. 9d. The fluctuations induced by vegetation are relatively small near the bed at the 10 cm measurement position. As shown in Fig. 9e, the variation in $\frac{{\sqrt {\overline{{u^{\prime 2} }} } }}{{\overline{U}}}$ and the velocity gradient becomes minimal in layers above 10 cm, compared to those near the channel bottom. In the non-vegetation area, momentum exchange in the low-velocity zone is more pronounced in both the lateral and vertical directions, emphasizing the impact of vegetation on flow.

### Verification of vegetation energy loss calculation methods

**Fig. 10:**
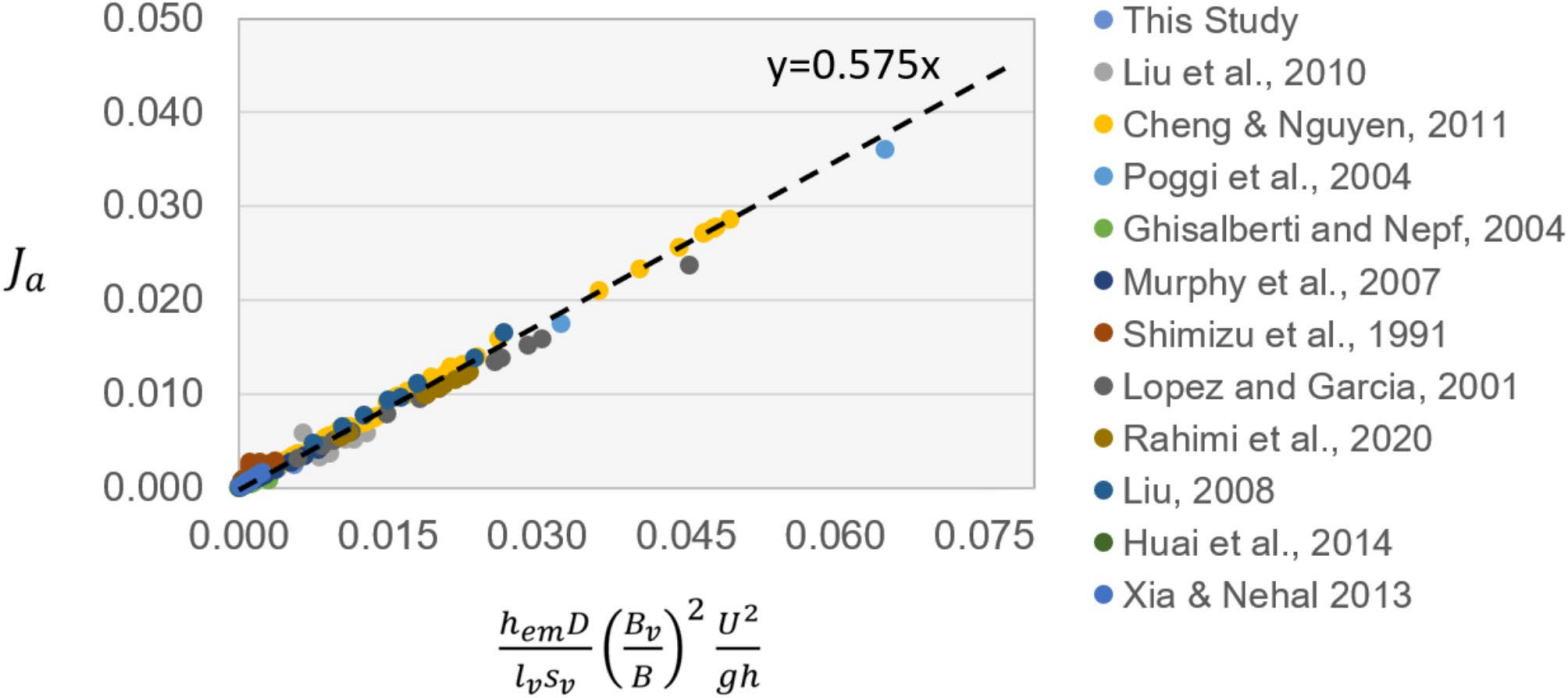
The relationship plot between ${J}_{a}$ and $\frac{{h}_{em}D}{{l}_{v}{s}_{v}}{\left(\frac{{B}_{v}}{B}\right)}^{2}\frac{{U}^{2}}{gH}$^17,28,31–39^.

To evaluate the feasibility of the new theoretical method for calculating energy loss in the vegetation patch, we analyzed a total of 262 experimental results, including those from the current study. The comparison is presented in Fig. 10. The observed value of ${J}_{a}$ is derived from the experiment by Huai et al.^31^. In the remaining experiments, ${J}_{a}$ was calculated using the observed ${U}_{v}$ data, as outlined below:

In the calculation of ${J}_{a}$, Eq. (16) can be used to rearrange Eq. (13) as follows:

22 $${F}_{Dm}={\phi }_{v}\frac{{2C}_{D}}{\pi }\frac{H}{D}{\rho U}_{v}^{2}$$

Based on Eq. (18), the energy gradient calculation method for energy loss due to vegetation is given by:

23 $${J}_{a}=\frac{{2C}_{D}}{\pi }\frac{{\phi }_{v}}{\left(1-{\phi }_{v}\right)}\frac{{U}_{v}^{2}}{g}$$

where, ${U}_{v}$ is the actual measured flow velocity at the vegetation patch.

In Fig. 10, the x-axis represents $\frac{{h}_{em}D}{{l}_{v}{s}_{v}}{\left(\frac{{B}_{v}}{B}\right)}^{2}\frac{{U}^{2}}{gH}$, calculated using Eq. (21), while the y-axis corresponds to ${J}_{a}$, which is either directly observed or derived from the observed ${U}_{v}$. The dataset includes 276 experimental data sets, covering both rigid and flexible vegetation experiments, with a Reynolds number ranging from 192 to 171,299. Figure 10 shows a regression slope of 0.575, which corresponds to ${C}_{D}$ based on Eq. (21).

In the experiment, the average submergence height of the vegetation, $\Delta {E}_{v}$, along with the vegetation density parameters ${s}_{v}$ and ${l}_{v}$, can be easily determined. Therefore, the observed dispersion in Fig. 10 is primarily influenced by variations in the drag coefficient, ${C}_{D}$.

The average measured value of ${C}_{D}$ is 1.15, leading to $\frac{{C}_{D}}{2}$ = 0.575, which confirms that Eq. (21) aligns well with the experimental data. These results indicate that ${C}_{D}$ is the primary factor affecting the precision of the vegetation energy gradient ${J}_{a}$ and head loss $\Delta {E}_{v}$. Consequently, future research will focus on developing methods for determining ${h}_{em}$ as well as the vegetation density parameters ${s}_{v}$ and ${l}_{v}$ for vegetation canopies.

### Discussion

Physical experiments and dynamic analysis are carried out in this study, which is based on the different distribution patterns of rigid vegetation. With validation from a large number of datasets (Fig. 10), the proposed method for calculating the energy loss due to flow resistance caused by vegetation patches can be applied to complex vegetation resistance calculations under natural conditions. The reason for this is that: (1) In cases where the angle of inclination of vegetation under the influence of flow is less than 10 degrees, it is equivalent to a 1.5% reduction in vegetation height (1 − cos10º = 0.015), which can be disregarded in engineering practice; (2) Regardless of how flexible vegetation tilts under the influence of flow, as long as the frontal area of flexible vegetation is determined, resistance can be calculated in a similar manner to that of rigid cylindrical objects.

This study calculates the resistance using the frontal area of vegetation. However, a challenge may arise when applying this method to field rivers: how can the frontal area of vegetation with diverse forms be determined? For example, how to properly generalize the height, diameter, and spacing of equivalent columns representing plant stems, branches, and leaves. This requires further systematic field observation. Moverover, this research was conducted under fixed bed conditions, but in natural rivers, resistance may also be influenced by factors such as erosion and deposition of sediment and changes in river bed morphology. These factors will be the focus of our future studies.

## Conclusions

This study proposes a method for calculating energy loss due to flow resistance caused by vegetation patches, based on the effective height of vegetation and energy principle. The method is applicable to complex vegetation resistance calculations under close-to-natural conditions. Three experiments were conducted on open-channel flows with uneven vegetation patch distribution to analyze flow characteristics, including vertical and lateral velocity distributions, turbulence intensity, and energy loss. The main findings are summarized as follows:

The presence of a vegetation patch elevates upstream water levels due to increased resistance, leading to higher flow velocities in the non-vegetated region. In the non-vegetated area, a low-velocity region is observed near the water surface, influenced by the flow interactions with the vegetated zone. Additionally, a low-velocity zone forms at 40%–60% of the relative depth within the transition area between vegetated and non-vegetated regions. This transition zone demonstrates significant vertical and horizontal momentum exchange.

An effective height (${h}_{e}$) was introduced to standardize vegetation height across varying submergence conditions. This parameter unified the computation methods for submerged and emergent vegetation within the experimental framework.

Theoretical formulas were derived to calculate water head loss ($\Delta {E}_{v}$) and energy gradient (${J}_{a}$) induced by the vegetation patch, enabling the assessment of energy loss in flows passing through mixed-height vegetation. In laboratory tests, parameters such as the average submerged vegetation height (${h}_{em}$), vegetation density (${s}_{v}$ and ${l}_{v}$), and resistance coefficient (${C}_{D}=1.15$) are easily measurable. However, in natural river channels, how to determine the effective height and frontal area of vegetation remains a challenge. Future research will focus on developing methods for accurately determining these parameters in natural environments.

## Data Availability

All data in this article were obtained through on-site measurements by the author. In addition, the data used to verify the results are indicated in the legend of Fig. 10, including: 10.1016/j.geomorph.2009.11.024 cited as Liu et al., 2010; 10.1029/2011WR010590 cited as Cheng, 2011; 10.1023/B:BOUN.0000016576.05621.73 cited as Poggi et al., 2004; 10.1029/2003WR002776 cited as Ghisalberti and Nepf, 2004; 10.1029/2006WR005229 cited as Murphy te al., 2007; Shimizu et al., 1991; 10.1061/(asce)0733-9429(2001)127:5(392) cited as López and García, 2001; 10.1016/j.advwatres.2020.103527 cited as Rahimi et al., 2020; 10.1029/2008JF001042 cited as Liu, 2008; 10.1016/j.advwatres.2014.04.001 cited as Huai et al., 2014; 10.3390/w5042080 cited as Xia& Nehal, 2013.
